# Add-on antiplatelet therapy in anticoagulated patients with atrial fibrillation

**DOI:** 10.1016/j.ijcha.2026.101907

**Published:** 2026-03-20

**Authors:** Yuki Matsuoka, Hitoshi Minamiguchi, Daisuke Sakamoto, Akihiro Sunaga, Katsuki Okada, Daisaku Nakatani, Tetsuhisa Kitamura, Takashi Kanda, Ryuta Watanabe, Kouichi Nagashima, Yoshiharu Higuchi, Yasuo Okumura, Yohei Sotomi, Yasushi Sakata

**Affiliations:** a Department of Cardiovascular Medicine, Osaka University Graduate School of Medicine, Osaka, Japan; b Cardiovascular Division, Osaka Keisatsu Hospital, Osaka, Japan; c Department of Medical Informatics, Osaka University Graduate School of Medicine, Osaka, Japan; d Department of Social and Environmental Medicine, Osaka University Graduate School of Medicine, Osaka, Japan; e Division of Cardiology, Department of Medicine, Nihon University School of Medicine, Tokyo, Japan

## Abstract

**Background:**

The impact of add-on antiplatelet therapy in patients with atrial fibrillation (AF) on oral anticoagulants (OAC) in real-world clinical practice remains to be investigated.

**Methods:**

We conducted DIRECT-Extend registry, a pooled analysis combining three large-scale real-world datasets of non-valvular AF patients treated with anticoagulation. We assessed clinical impacts of the add-on antiplatelet therapy using the inverse-probability-of-treatment weighting methods. Antiplatelet therapy included aspirin, P2Y12 inhibitors, and cilostazol (dual therapy included). The primary ischemic endpoint was a composite of all-cause death, ischemic stroke, systemic embolism, and myocardial infarction. The primary bleeding endpoint was any bleeding, defined as a composite of major bleeding and clinically relevant non-major bleeding according to the criteria of the International Society on Thrombosis and Hemostasis.

**Results:**

A total 7387 eligible patients (excluding those within 1 year after percutaneous coronary intervention (PCI) or coronary artery bypass grafting (CABG)) were divided into two groups: the OAC alone group (N = 6096) and the OAC + APT group (treated with both OAC and antiplatelet therapy, N = 1291). The median follow-up period was 1012 [404, 1344] days. The risk for both the primary ischemic and bleeding endpoint was higher in patients in OAC + APT group than those in OAC alone group (ischemic endpoint, weighted hazard ratio (wHR): 1.28, 95%CI [1.17 – 1.40], p < 0.001; bleeding endpoint, wHR: 1.26 [1.19–1.33], p < 0.001).

**Conclusion:**

The large-scale real-world data demonstrated that, in AF patients treated with OAC, add-on antiplatelet therapy was associated with a higher risk for both ischemic and bleeding endpoints. These findings may not be generalizable to the early post-PCI/CABG period.

## Graphical abstract

**Figure:**
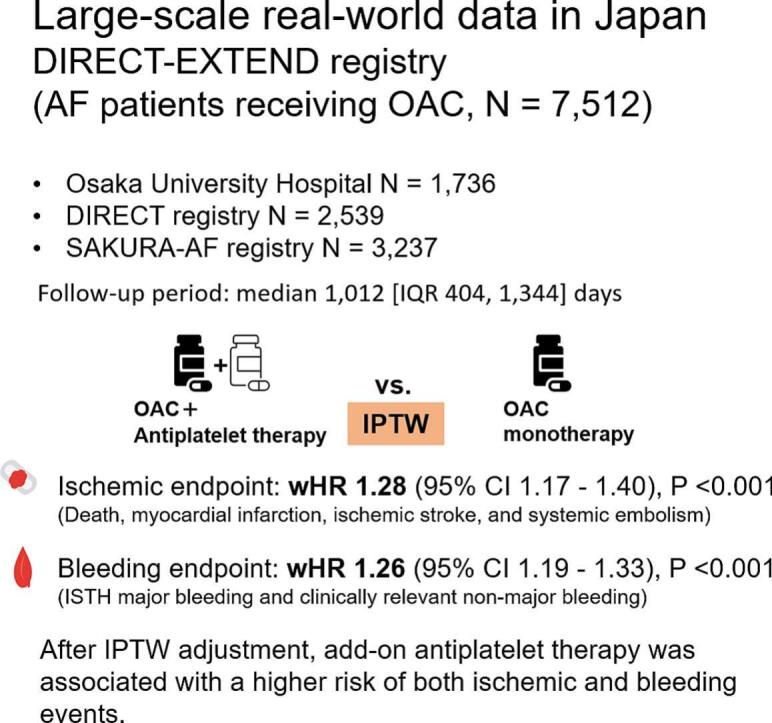

## 1 Introduction

Patients with atrial fibrillation (AF) are recommended to receive oral anticoagulants (OAC) to prevent ischemic stroke. Patients with atherosclerotic disease are recommended to receive antiplatelet therapy to prevent ischemic events. The population requiring both antiplatelet therapy and OAC often overlaps. However, the combination of these therapies increases the risk of bleeding [1], [2]. The AFIRE trial, which investigated the use of antithrombotic therapy in patients with AF and stable coronary artery disease, found that rivaroxaban monotherapy was noninferior to combination therapy of rivaroxaban and antiplatelet therapy in terms of efficacy and superior in terms of safety [3]. According to the recent guidelines, OAC monotherapy is recommended for patients with AF and atherosclerotic diseases [4], [5], [6]. Nevertheless, add-on antiplatelet therapy is still used in routine practice, often driven by perceived ischemic risk or comorbidity burden. Contemporary, population-level real-world data quantifying the associated risks, particularly for both ischemic and bleeding outcomes, remain limited. In this study, we aimed to evaluate the impact of add-on antiplatelet therapy among anticoagulated patients with AF in the real-world clinical practice.

## 2 Methods

### 2.1 Study design and study population

We conducted DIRECT-Extend registry (UMIN000050585), a pooled analysis combining three large-scale real-world datasets of non-valvular AF patients treated with anticoagulation [DIRECT registry (N = 2543), SAKURA-AF registry (N = 3268), and Osaka University Hospital registry (N = 1742)]. Patients who visited or were admitted to the study institutions (Osaka University Hospital, Osaka Keisatsu Hospital, and 63 facilities participating in SAKURA-AF) and who were newly prescribed anticoagulants for non-valvular AF were enrolled.

1) In the registry of Osaka University Hospital, we collected data including patient demographics, comorbidities, medication history, laboratory and echocardiographic data, and outcome information retrospectively from electronic medical records. This registry included patients prescribed direct oral anticoagulants (DOACs) between March 2011 and December 2021. 2) The DIRECT registry is a single-center registry of non-valvular AF patients on DOACs at Osaka Keisatsu Hospital from June 2011 to November 2017. In addition to the original dataset, we further collected additional patient data and extended the follow-up period up to November 2023 for this pooled analysis. 3) The SAKURA-AF registry included 3268 patients with non-valvular AF treated with any OAC including warfarin for stroke prevention in contrast to the former two registries. Patients were enrolled at any of 63 participating institutions (2 cardiovascular centers, 13 affiliated hospitals or community hospitals, and 48 private clinics) in the Tokyo area between September 2013 and December 2015 and followed up until December 2017.

We integrated these three registry datasets and unified the outcomes and variables to create a larger registry of AF patients on OAC, the DIRECT-Extend registry. The registry complies with all the principles of the Declaration of Helsinki, and the study protocol was approved by the Ethical Committee of each institution.

In this analysis, we investigated the impact of the add-on antiplatelet therapy on ischemic and bleeding endpoints in AF patients with OAC. We divided all patients into two groups based on the concomitant use of antiplatelet agents: the OAC alone group (treated with OAC monotherapy) and the OAC + APT group (treated with both OAC and antiplatelet therapy). We also conducted subgroup analysis for the presence of vascular disease. Vascular disease was defined as the presence of any of followings: coronary artery disease, myocardial infarction, aortic plaque, prior history of percutaneous coronary intervention (PCI) or coronary artery bypass grafting (CABG), and peripheral artery disease [7]. Antiplatelet therapy was defined as the use of one or more of the following antiplatelet agents: aspirin, clopidogrel, prasugrel, ticlopidine, and cilostazol including dual antiplatelet therapy. Patients who had undergone percutaneous coronary intervention (PCI) or coronary artery bypass grafting (CABG) within the previous year were excluded due to the heightened thrombotic risk associated with the acute phase following these procedures [4].

### 2.2 Study endpoints

The primary ischemic endpoint was a composite of all-cause death, ischemic stroke, systemic embolism, and myocardial infarction. The primary bleeding endpoint was any bleeding, defined as a composite of major bleeding and clinically relevant non-major bleeding, according to the criteria of the International Society on Thrombosis and Hemostasis. Each individual outcome is analyzed separately as a secondary endpoint.

### 2.3 Statistical analysis

All statistical analyses were performed with R software (V.4.3.1; R Foundation for Statistical Computing, Vienna, Austria). P values less than 0.05 were considered statistically significant. Categorical variables were expressed as counts (percentages) and compared with the chi-squared test or Fisher’s exact test. Continuous variables were expressed as mean (SD) or median (IQR) and were compared using analysis of student’s *t*-test or Kruskal-Wallis test, as appropriate. Differences in survival curves between the patient groups were estimated using the Kaplan-Meier method and analyzed using the log-rank test (‘jskm’ package). Since the exclusion of cases with missing data can cause bias in analysis and loss of power in detecting statistical differences, missing values in the variables used in the following analyses were imputed by random forest imputation using the ‘miss-Forest’ package prior to analysis. After imputation, we estimated propensity scores by fitting a multivariable logistic regression model with the following variables: age, female sex, body mass index, creatinine clearance, history of stroke or transient ischemic attack, vascular disease, diabetes mellitus, hypertension, dyslipidemia, history of bleeding, liver dysfunction, type of OAC, hemoglobin, platelet count, and the registry the patient was included in. These variables were selected based on the component of CHA_2_DS_2_-VASc score and ORBIT score as well as variables considered clinically important [8], [9]. We established weighted Cox proportional hazards regression models with inverse-probability-of-treatment weighting (IPTW) to reduce the impact of measured confounding inherent to the observational design. To account for potential variability across the three integrated registries, we incorporated a mixed-effects model, treating the registry as a random effect. To assess covariate balance, we examined absolute standardized mean differences (SMDs) before and after weighting. As a heuristic, an absolute SMD < 0.10 was considered indicative of negligible imbalance [10], [11]. IPTW was primarily implemented using unstabilized weights (1/PS for the OAC + APT group and 1/[1 − PS] for the OAC-alone group). As diagnostics and sensitivity analyses, we additionally evaluated stabilized weights and truncation at the 1st/99th percentiles, and reanalyzed the primary endpoints using these alternative weighting specifications. We also performed sensitivity analyses using an alternative ischemic composite excluding myocardial infarction and after excluding the SAKURA-AF registry (with propensity scores and weights re-estimated in the restricted cohort). As a sensitivity analysis for unmeasured confounding, we calculated E-values for the primary endpoints estimates obtained from the IPTW-weighted Cox proportional hazards models. E-values were calculated for both the point estimates and the confidence interval bounds closest to the null. The results are summarized as weighted hazard ratios (wHRs) and 95% confidence interval (CI). The proportional hazards assumption for the primary endpoints was confirmed by Schoenfeld residuals.

## 3 Results

### 3.1 Study patients

Supplemental Fig. 1 illustrates the patient flowchart. Of 7512 patients, 125 patients were excluded due to PCI or CABG within the past one year, leaving 7387 eligible patients. These were divided into two groups by antiplatelet therapy use: the OAC alone group (N = 6096; 82.5%) and the OAC + APT group (N = 1291; 17.5%). The patients’ backgrounds are shown in Table 1. In the OAC + APT group, aspirin was the predominant antiplatelet agent (77.1%), followed by clopidogrel (22.1%), with smaller proportions of cilostazol (7.8%), ticlopidine (1.5%), and prasugrel (1.2%). We estimated the impact of add-on antiplatelet therapy using the IPTW method. Supplemental Fig. 2 shows the distributions of propensity scores and unstabilized IPTW weights in the two groups. The post-weighted hypothetical patients’ backgrounds are shown in Table 2. The SMD of each variable was generally low. The median follow-up period was 1012 [404, 1344] days.

**Table 1:** Unweighted baseline characteristics.

	OAC alone	OAC + APT	SMD	Missing (%)
N	6096	1291		
Registry			0.194	0
SAKURA-AF	2761 (45.3)	476 (36.9)		
DIRECT registry	1948 (32.0)	523 (40.5)		
Osaka University Hospital	1387 (22.8)	292 (22.6)		
Age	72.0 [65.0, 78.0]	75.0 [69.0, 81.0]	0.377	0
Female	1957 (32.1)	343 (26.6)	0.122	0
Body mass index, kg/m^2^	23.45 [20.94, 26.17]	23.85 [21.31, 26.57]	0.057	1.5
Type of AF			0.132	0
Paroxysmal	2996 (49.1)	670 (51.9)		
Persistent	1139 (18.7)	184 (14.3)		
Long-standing persistent	1721 (28.2)	369 (28.6)		
Unknown	240 (3.9)	68 (5.3)		
History of stroke or transient ischemic attack	903 (14.8)	424 (32.8)	0.433	0
Hypertension	4017 (65.9)	1071 (83.0)	0.399	0
Diabetes	1309 (21.5)	471 (36.5)	0.335	0
Dyslipidemia	2617 (42.9)	831 (64.4)	0.441	0
History of heart failure	1598 (26.2)	424 (32.9)	0.146	0
Vascular disease	532 (8.7)	732 (56.7)	1.190	0
History of CAD	189 (3.1)	514 (39.8)	1.000	0
History of bleeding	467 (7.7)	179 (13.9)	0.201	0
CHA_2_DS_2-_VASc score	4.0 [3.0, 5.0]	5.0 [4.0, 7.0]	0.871	0.1
HAS-BLED score	2.0 [2.0, 3.0]	4.0 [3.0, 5.0]	1.433	2.8
Type of OAC			0.147	0
Dabigatran	1138 (18.7)	213 (16.5)		
Rivaroxaban	1598 (26.2)	283 (21.9)		
Apixaban	1332 (21.9)	347 (26.9)		
Edoxaban	754 (12.4)	161 (12.5)		
Warfarin	1274 (20.9)	287 (22.2)		
Aspirin	0 (0.0)	995 (77.1)	2.593	0
Clopidogrel	0 (0.0)	285 (22.1)	0.753	0
Prasugrel	0 (0.0)	15 (1.2)	0.153	0
Ticlopidine	0 (0.0)	20 (1.5)	0.177	0
Cilostazol	0 (0.0)	101 (7.8)	0.412	0
Beta blocker	2758 (45.2)	694 (53.8)	0.171	0
Calcium channel blocker	1501 (24.6)	473 (36.6)	0.263	0
Hemoglobin, g/dL	13.7 [12.5, 14.9]	13.3 [11.9, 14.6]	0.228	4.0
Platelet, 10^3^/µL	194.0 [162.0, 231.0]	186.0 [156.0, 224.0]	0.103	4.3
Creatinine, mg/dL	0.85 [0.72, 1.01]	0.93 [0.78, 1.12]	0.273	3.1
Estimated glomerular filtration rate, mL/min/1.73 m^2^	63.1 [52.3, 74.5]	57.8 [47.0, 68.9]	0.319	3.1
Creatinine clearance, mL/min	66.0 [50.3, 84.6]	58.1 [43.2, 74.2]	0.357	3.4
BNP, pg/mL	113.6 [52.9, 224.7]	120.8 [59.1, 231.6]	0.029	62.0
NT-proBNP, pg/mL	629.8 [227.0, 1387.5]	757.4 [347.8, 1537.0]	0.010	59.7
Albumin, g/dL	4.0 [3.6, 4.3]	3.9 [3.5, 4.2]	0.199	67.3
Low-density lipoprotein cholesterol, mg/dL	104.0 [85.0, 124.0]	92.0 [76.0, 112.0]	0.361	19.0
Total bilirubin, mg/dL	0.7 [0.5, 0.9]	0.6 [0.5, 0.9]	0.141	49.6
Aspartate aminotransferase, IU/L	24.0 [20.0, 30.0]	23.0 [19.0, 30.0]	0.047	4.3
Alanine aminotransferase, IU/L	19.0 [14.0, 27.0]	18.0 [13.0, 25.0]	0.068	4.6

Data are expressed as median [IQR] or number (percentage). OAC alone group: treated with oral anticoagulants alone; OAC + APT group: treated with both oral anticoagulants and antiplatelet therapy. Abbreviations; SMD, standardized mean difference; OAC, oral anticoagulants; BNP, brain natriuretic peptide, NT-proBNP, N-terminal pro-brain natriuretic peptide.

**Table 2:** Baseline Characteristics of Study Population after Inverse Probability of Treatment Weighting.

	OAC alone	OAC + APT	SMD
N	7453	7044	
Age	73.0 [66.0, 79.0]	74.0 [67.0, 80.0]	0.022
Female	2344 (31.4)	2496 (35.4)	0.085
Body mass index, kg/m^2^	23.5 [21.0, 26.2]	23.5 [21.3, 26.0]	0.015
Creatinine clearance, mL/min	64.2 [49.4, 82.8]	62.5 [47.1, 81.2]	0.056
History of stroke or transient ischemic attack	1446 (19.4)	1556 (22.1)	0.066
Hypertension	5144 (69.0)	4817 (68.4)	0.014
Diabetes	1810 (24.3)	1739 (24.7)	0.009
Dyslipidemia	3479 (46.7)	3264 (46.3)	0.007
History of bleeding	672 (9.0)	784 (11.1)	0.071
Vascular disease	1334 (17.9)	1289 (18.3)	0.010
Liver dysfunction	1023 (13.7)	990 (14.1)	0.010
Type of OAC			0.100
Dabigatran	1344 (18.0)	1295 (18.4)	
Rivaroxaban	1883 (25.3)	1522 (21.6)	
Apixaban	1721 (23.1)	1688 (24.0)	
Edoxaban	946 (12.7)	1056 (15.0)	
Warfarin	1559 (20.9)	1483 (21.1)	
Hemoglobin, g/dL	13.6 [12.4, 14.8]	13.4 [12.1, 14.8]	0.085
Platelet, 10^3^/µL	194.0 [163.0, 228.0]	191.0 [159.0, 227.0]	0.033
Registry			0.031
SAKURA-AF	3232 (43.4)	2953 (41.9)	
DIRECT registry	2545 (34.2)	2444 (34.7)	
Osaka University Hospital	1675 (22.5)	1647 (23.4)	

Data are expressed as median [IQR] or number (percentage). OAC alone group: treated with oral anticoagulants alone; OAC + APT group: treated with both oral anticoagulants and antiplatelet therapy. Weighted N represents the sum of IPTW weights (pseudo-population), not the number of observed patients. Abbreviations; SMD, standardized mean difference.

## 4 The prognostic impact of add-on antiplatelet therapy

**Fig. 1:**
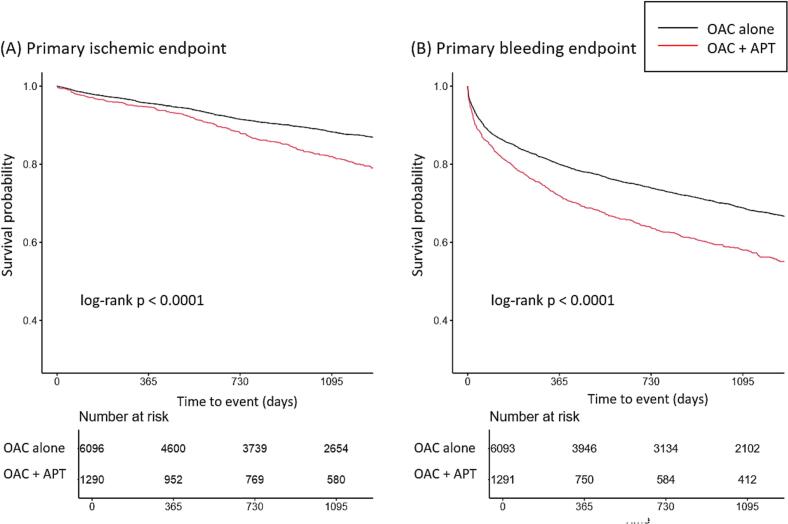
Kaplan-Meier curves for primary endpoints. Survival analysis using the Kaplan-Meier method for (A) the primary ischemic endpoint (a composite of all-cause death, ischemic stroke, systemic embolism, and myocardial infarction) and (B) the primary bleeding endpoint (any bleeding; a composite of major bleeding and clinically relevant non-major bleeding, according to the criteria of the International Society on Thrombosis and Hemostasis) is illustrated. OAC alone group: treated with oral anticoagulants alone; OAC + APT group: treated with both oral anticoagulants and antiplatelet therapy.

Kaplan-Meier curves are illustrated in Fig. 1. Patients in the OAC + APT group had a significantly higher incidence of both the primary ischemic and bleeding endpoints compared to the OAC alone group (ischemic endpoint, log-rank p < 0.001; bleeding endpoint, log-rank p < 0.001). Cox proportional hazards models with IPTW showed a significantly increased risk for both the primary ischemic and bleeding endpoints in the OAC + APT group compared to the OAC alone group (Table 3, ischemic endpoint, wHR: 1.28, 95% CI: 1.17 – 1.40, p < 0.001; bleeding endpoint, wHR: 1.26, 95% CI: 1.19 – 1.33, p < 0.001). In an E-value sensitivity analysis for the primary endpoints, the E-values for the point estimates and the confidence interval bounds closest to the null were 1.88 and 1.62 for the ischemic endpoint, and 1.83 and 1.67 for the bleeding endpoint, respectively. For secondary endpoints, the OAC + APT group showed a higher risk for systemic embolism (wHR: 1.40, 95% CI: 1.05 – 1.86, p = 0.022) and myocardial infarction (wHR: 1.68, 95% CI: 1.45 – 1.97, p < 0.001). Additionally, the risk of both major bleeding and clinically relevant non-major bleeding were significantly greater in the OAC + APT group compared to the OAC alone group (Table 3, major bleeding, wHR: 1.13, 95% CI: 1.01 – 1.28, p = 0.035; clinically relevant non-major bleeding, wHR: 1.29, 95% CI: 1.22 – 1.37, p < 0.001).

**Table 3:** Clinical impact of add-on antiplatelet therapy for ischemic and bleeding events in overall population.

	Event number		Event rate (/100 person-years)				
	OAC alone	OAC + APT	OAC alone	OAC + APT	wHR	95% CI	P value
	N = 6096	N = 1291	N = 6096	N = 1291			
Primary ischemic endpoint (a composite of all-cause death, ischemic stroke, systemic embolism, and myocardial infarction)	635	231	3.86	6.49	1.28	1.17–––1.40	<0.001
All-cause death	344	123	2.03	3.30	1.13	1.00–––1.27	0.055
Ischemic stroke	169	46	1.01	1.25	1.09	0.91––1.31	0.332
Systemic embolism	62	28	0.37	0.76	1.40	1.05–––1.86	0.022
Myocardial infarction	207	81	1.24	2.23	1.68	1.45–––1.97	<0.001
Primary bleeding endpoint (Any bleeding)	1794	524	13.77	20.88	1.26	1.19–––1.33	<0.001
Major bleeding	406	133	2.48	3.76	1.13	1.01–––1.28	0.035
Clinically relevant non-major bleeding	1613	484	12.15	18.83	1.29	1.22–––1.37	<0.001

Event rates are crude incidence rates per 100 person-years (unweighted). wHRs were estimated using IPTW-weighted Cox models. P for interaction was derived from a treatment-by-vascular disease interaction term. Abbreviations: wHR, weighted hazard ratio; CI, confidence interval.

We evaluated IPTW diagnostics, including the distributions of propensity scores and unstabilized weights (Supplemental Fig. 2) and summaries of weight distributions and effective sample size (Supplemental Table 1). Sensitivity analyses using stabilized weights with 1st/99th percentile truncation yielded consistent results for both primary endpoints (Supplemental Table 2a, ischemic endpoint, wHR: 1.34, 95% CI: 1.14 – 1.57, p < 0.001; bleeding endpoint, wHR: 1.28, 95% CI: 1.15 – 1.42, p < 0.001). In a sensitivity analysis using an ischemic composite excluding myocardial infarction (all-cause death, ischemic stroke, and systemic embolism), add-on antiplatelet therapy remained associated with a higher risk (wHR 1.15, 95% CI 1.04–1.27; p = 0.008, Supplemental Table 2b). In an additional sensitivity analysis excluding the SAKURA-AF registry, the association with the primary ischemic endpoint was attenuated and no longer statistically significant (wHR 1.18, 95% CI 0.98–1.28; p = 0.107), whereas the association with the primary bleeding endpoint remained significant (wHR 1.27, 95% CI 1.19–1.35; p < 0.001, Supplemental Table 2c). In patients who experienced both bleeding and ischemic events, ischemic events tended to cluster shortly after bleeding events (Supplemental Fig. 3).

### 4.1 Subgroup analysis

**Fig. 2:**
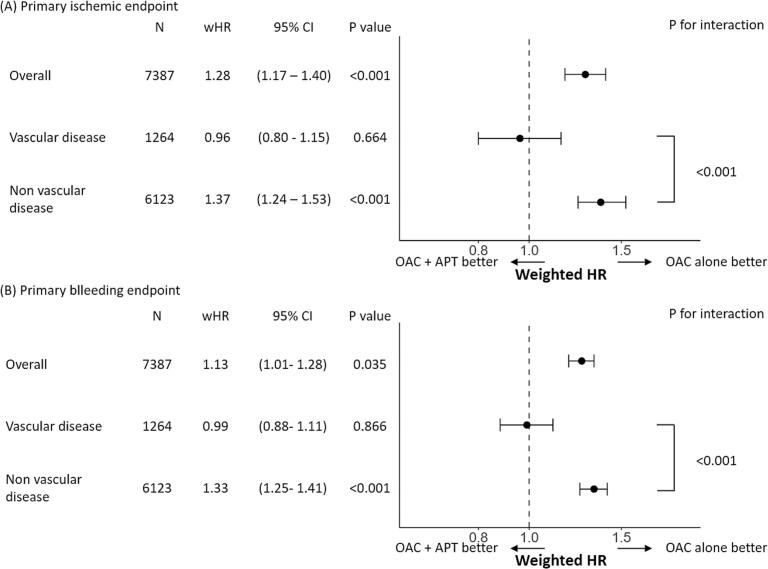
Clinical impact of add-on antiplatelet therapy for ischemic and bleeding events in subgroup analysis. For both the ischemic and bleeding endpoint, the risk was comparable between the OAC alone group and the OAC + APT group in patients with vascular disease, whereas it was significantly higher in the OAC + APT group compared to the OAC alone group in patients without vascular disease (p for interaction < 0.001). Abbreviations, wHR, weighted hazard ratio; CI, confidence interval; OAC alone group: treated with oral anticoagulants alone; OAC + APT group: treated with both oral anticoagulants and antiplatelet therapy.

We conducted subgroup analysis for the presence of vascular disease. The post-weighted hypothetical patients’ backgrounds of each subgroup are shown in Supplemental Table 3. The Kaplan-Meier curves are illustrated in Supplemental Fig. 4. Among patients with vascular disease, the risk of both ischemic and bleeding endpoints was similar between the OAC-alone group and the OAC + APT group (log-rank p = 0.60 for both endpoints). In contrast, among patients without vascular disease, those in the OAC + APT group demonstrated a significantly higher risk for both the primary ischemic and bleeding endpoints compared to the OAC alone group (p < 0.001 for both endpoints). The results of the Cox proportional hazards model with IPTW are summarized in Fig. 2. For the ischemic endpoint, the risk was comparable between the OAC alone group and the OAC + APT group in patients with vascular disease (wHR: 0.96, 95% CI: 0.80 – 1.15, p = 0.664), whereas it was significantly higher in the OAC + APT group compared to the OAC alone group in patients without vascular disease (wHR: 1.37, 95% CI: 1.24–––1.53, p < 0.001; p for interaction < 0.001). For the bleeding endpoint, the risk was comparable between the two groups in patients with vascular disease (wHR: 0.99, 95% CI: 0.88 – 1.33, p = 0.866), whereas it was significantly higher in the OAC + APT group compared to the OAC alone group in patients without vascular disease (wHR: 1.33, 95% CI: 1.25–––1.41, p < 0.001; p for interaction < 0.001). The impact for each secondary endpoint is summarized in Supplemental Table 4.

## 5 Discussion

Main findings of the present study, based on a large-scale real-world cohort, can be summarized as follows: 1) the risk of both the primary ischemic and bleeding endpoints was significantly higher in the OAC + APT group than in the OAC alone group; 2) for the secondary endpoints, the risk of myocardial infarction, systemic embolism and clinically relevant non-major bleeding was higher in patients in the OAC + APT group compared to those in the OAC alone group; and 3) in exploratory subgroup analyses stratified by vascular disease status, add-on antiplatelet therapy did not show clear benefit among patients with vascular disease, whereas it was associated with higher risks of both primary endpoints among those without vascular disease. Despite available randomized trial evidence, contemporary large-scale real-world data from Japan evaluating add-on antiplatelet therapy in anticoagulated AF patients are limited. Our pooled registry analysis provides population-level estimates from routine practice and broadly reinforces guideline-concordant management.

## 6 Combination of anticoagulant and antiplatelet therapy in AF patients

In the AFIRE trial (N = 2240), which investigated antithrombotic strategies in patients with AF and stable coronary artery disease, rivaroxaban monotherapy was noninferior to combination therapy for ischemic endpoints and superior with respect to major bleeding [3]. The EPIC-CAD trial (N = 1040) recently showed that edoxaban monotherapy was associated with a lower risk of a composite of death from any cause, myocardial infarction, stroke, systemic embolism, unplanned urgent revascularization, or major bleeding or clinically relevant nonmajor bleeding than dual antithrombotic therapy in patients with AF and stable coronary disease [12]. The AQUATIC trial (N = 872) recently showed that, in patients with chronic coronary syndrome who had undergone prior coronary stenting (>6 months) and were receiving long-term oral anticoagulation, the addition of aspirin led to a higher risk of a composite of cardiovascular death, myocardial infarction, stroke, systemic embolism, coronary revascularization, or acute limb ischemia, as well as higher risks of major bleeding and death from any cause, than placebo [13]. Collectively, these randomized controlled trials support the clinical benefits of OAC monotherapy. A recent review summarizing randomized trials and *meta*-analyses emphasizes individualized antithrombotic optimization in patients with atrial fibrillation undergoing PCI, given the consistent increase in bleeding with more intensive regimens [14]. In this context, we evaluated real-world outcomes associated with add-on antiplatelet therapy in Japanese registry populations. In our large-scale real-world cohort, which included patients beyond those with coronary artery disease, we found the risk for both the ischemic and bleeding endpoints were significantly higher among patients treated with OAC plus antiplatelet therapy than those with OAC monotherapy. Antiplatelet agents are primarily prescribed for the prevention of ischemic events. However, in this study, patients receiving antiplatelet therapy not only had a higher risk of bleeding events but also a higher risk of ischemic events compared to those on OAC monotherapy. Previous studies have reported that patients with AF who experience bleeding events subsequently face an elevated risk of ischemic events [15]. Unexpected discontinuation of antithrombotic therapy due to bleeding events has been implicated as a contributing factor to the increased risk of ischemic events [16]. Consistently, our data suggested that ischemic events tended to cluster after bleeding events (Supplemental Fig. 3).

Subgroup analysis did not show any benefit of the add-on antiplatelet therapy for patients with vascular disease in our real-world cohort, consistent with findings from previous randomized trials. Using IPTW, baseline characteristics were well balanced between groups, and we observed no meaningful difference in bleeding events with add-on antiplatelet therapy. This neutral bleeding signal, which contrasts with several randomized trials, may reflect differences in the underlying bleeding risk profile of our population. Notably, patients with vascular disease in our registry had a higher baseline bleeding risk than those enrolled in the AFIRE trial (the proportion of patients with a history of bleeding after weighting: 17.0% vs 2.7%, Supplemental Table 3). In such a high-risk setting, the incremental bleeding hazard attributable to antiplatelet therapy may be attenuated or less readily detectable. In summary, the previous randomized trials and our large-scale real-world data consistently did not show the clinical benefit of add-on antiplatelet therapy in AF patients with vascular disease such as chronic phase of coronary artery disease. As to the acute phase (within 1 year of PCI/CABG), a prospective study evaluating short-term dual therapy followed by DOAC monotherapy in AF patients undergoing PCI is currently in progress [17].

## 7 Combination of anticoagulant and antiplatelet therapy in AF patients without vascular disease

Antiplatelet therapy is widely used for the prevention of atherosclerotic events. Previous *meta*-analyses have shown that antiplatelet therapy in primary prevention reduces ischemic events, but increases bleeding events [18]. In Japan, a study evaluating the efficacy of aspirin for primary prevention in high-risk patients (e.g., those with diabetes mellitus, hypertension, and dyslipidemia) without established vascular disease reported a reduction in ischemic events but an increase in bleeding events [19]. Accordingly, recent guidelines do not recommend the routine use of antiplatelet therapy for primary prevention. However, some patients in routine clinical practice receive this therapy. Our subgroup analysis showed that adding antiplatelet agents was associated with higher ischemic and bleeding events among patients without vascular disease. These bleeding complications may have contributed to subsequent ischemic events by necessitating interruptions in antithrombotic therapy and by triggering compensatory upregulation of hemostatic pathways [15], [16]. Therefore, in AF patients using anticoagulants without vascular disease, concomitant use of antiplatelet agents for primary prevention would not be recommended.

## 8 Limitation

Several limitations should be acknowledged. First, outcome definitions differed across the three registries. Specifically, the SAKURA-AF registry includes unstable angina in the definition of myocardial infarction; therefore, MI-related results should be interpreted with caution. We accounted for between-registry heterogeneity by including registry in the propensity score model and using a mixed-effects Cox model with registry as a random effect, and the overall conclusions were consistent in a sensitivity analysis excluding myocardial infarction. Second, while patients who underwent PCI or CABG within one year were excluded, the SAKURA-AF registry lacked detailed information regarding revascularization before enrollment, making it impossible to exclude such patients. This limitation may have preferentially retained higher thrombotic-risk patients in the OAC + APT group and contributed to confounding by indication. Consistently, in a sensitivity analysis excluding the SAKURA-AF registry, the association with the primary ischemic endpoint was attenuated, whereas the association with the primary bleeding endpoint remained significant. In addition, other clinically legitimate indications for antiplatelet therapy (e.g., recent MI or medically managed ACS without PCI, or peripheral interventions for PAD) were not consistently captured across registries. Therefore, we could not determine the rationale for antiplatelet prescription at the individual level or fully exclude patients with such indications, which may have introduced residual confounding. Third, changes in antithrombotic therapy during follow-up (initiation, discontinuation, or crossover) were not consistently captured across the registries. Accordingly, treatment was defined at baseline, and the observed long-term associations may be biased due to time-varying exposure and misclassification during follow-up. This limitation is particularly relevant because antithrombotic therapy may change after events, which could influence subsequent outcomes. In addition, antiplatelet regimens were aspirin-dominant and included P2Y12 inhibitors and cilostazol, with some patients receiving more than one antiplatelet agent. Ticagrelor use was not captured in the included registries; therefore, agent-specific comparisons were not feasible and generalizability to settings where ticagrelor is commonly used may be limited. Fourth, although IPTW balanced measured covariates, treatment selection bias and residual confounding may still have influenced the observed associations because some clinically relevant factors were unavailable (e.g., coronary disease severity and frailty), or had substantial missingness (e.g., gastroprotective therapy) and therefore could not be fully accounted for. In line with this concern, E-value analyses for the primary endpoints suggested that unmeasured confounding of modest strength could potentially explain away the observed associations, and the findings should therefore be interpreted as associative rather than causal. Fifth, subgroup analyses (including the vascular disease subgroup) should be interpreted with caution because of subgroup size imbalance and limited precision. These analyses were exploratory and not powered for modest subgroup differences; therefore, the absence of an observed difference should not be interpreted as evidence of no treatment effect. Differences from randomized trials (e.g., AFIRE) may also reflect differences in patient selection, risk profile, and treatment context. Finally, since this study was based on Japanese registries, caution is warranted when generalizing the findings to other populations or regions, given potential ethnic and healthcare-system differences in bleeding risk and antithrombotic use.

## 9 Conclusion

The large-scale real-world data in Japan demonstrated that, in AF patients treated with OAC, add-on antiplatelet therapy was associated with a higher risk for both ischemic and bleeding endpoints. These findings may not be generalizable to patients in the early post-PCI/CABG period, in whom antithrombotic management and baseline risk profiles differ substantially.

## 10 Authorship statement

All authors take responsibility for all aspects of the reliability and freedom from bias of the data presented and their discussed interpretation.

## Sources of funding

This study was supported by a grant from JSPS 10.13039/501100001691KAKENHI Grant Number JP22K16073.

## Conflict of Interest

Akihiro Sunaga has received personal fees from Daiichi Sankyo. Takashi Kanda has received personal fees from Boehringer Ingelheim, Daiichi Sankyo. Hitoshi Minamiguchi has received personal fees from Bayer, Boehringer Ingelheim, Bristole-Myers Squibb, Daiichi Sankyo, Pfizer Pharmaceuticals. Yasuo Okumura received research grants unrelated to this study from Nippon Boehringer Ingelheim, remuneration from Daiichi-Sankyo, Bayer Healthcare, Bristol-Myers Squibb. Yohei Sotomi has received grants from Bristol-Myers Squibb, and personal fees from Bayer, Boehringer Ingelheim, Bristole-Myers Squibb, Daiichi Sankyo, and Pfizer Pharmaceuticals. Yasushi Sakata has received grants from Bristol-Myers Squibb Company, Bayer AG, and Boehringer Ingelheim, and personal fees from Bristol-Myers Squibb Company, Pfizer Inc, Bayer AG, Daiichi Sankyo Company, and Boehringer Ingelheim. The other authors have nothing to disclose.

## CRediT authorship contribution statement

**Yuki Matsuoka:** Writing – review & editing, Writing – original draft, Visualization, Validation, Resources, Project administration, Methodology, Investigation, Formal analysis, Data curation, Conceptualization. **Hitoshi Minamiguchi:** Writing – review & editing, Methodology, Conceptualization. **Daisuke Sakamoto:** Writing – review & editing, Supervision. **Akihiro Sunaga:** Writing – review & editing, Supervision. **Katsuki Okada:** Writing – review & editing, Supervision. **Daisaku Nakatani:** Writing – review & editing, Supervision, Conceptualization. **Tetsuhisa Kitamura:** Writing – review & editing, Visualization, Conceptualization. **Takashi Kanda:** Writing – review & editing. **Ryuta Watanabe:** Writing – review & editing. **Kouichi Nagashima:** Writing – review & editing. **Yoshiharu Higuchi:** Writing – review & editing. **Yasuo Okumura:** Writing – review & editing. **Yohei Sotomi:** Writing – review & editing, Writing – original draft, Validation, Supervision, Software, Resources, Project administration, Investigation, Funding acquisition, Data curation. **Yasushi Sakata:** Writing – review & editing.

## Declaration of competing interest

The authors declare the following financial interests/personal relationships which may be considered as potential competing interests: Yohei Sotomi reports financial support was provided by JPSS KAKENHI. Akihiro Sunaga reports a relationship with Daiichi Sankyo Inc that includes: speaking and lecture fees. Takashi Kanda reports a relationship with Boehringer Ingelheim that includes: speaking and lecture fees. Takashi Kanda reports a relationship with Daiichi Sankyo Inc that includes: speaking and lecture fees. Hitoshi Minamiguchi reports a relationship with Daiichi Sankyo Inc that includes: speaking and lecture fees. Hitoshi Minamiguchi reports a relationship with Bayer that includes: speaking and lecture fees. Hitoshi Minamiguchi reports a relationship with Boehringer Ingelheim that includes: speaking and lecture fees. Hitoshi Minamiguchi reports a relationship with Bristol Myers Squibb that includes: speaking and lecture fees. Hitoshi Minamiguchi reports a relationship with Pfizer that includes: speaking and lecture fees. Yasuo Okumura reports a relationship with Nippon Boehringer Ingelheim that includes: funding grants. Yasuo Okumura reports a relationship with Daiichi Sankyo Inc that includes: speaking and lecture fees. Yasuo Okumura reports a relationship with Bayer Healthcare that includes: speaking and lecture fees. Yasuo Okumura reports a relationship with Bristol Myers Squibb that includes: speaking and lecture fees. Yohei Sotomi reports a relationship with Bristol-Myers Squibb that includes: funding grants and speaking and lecture fees. Yohei Sotomi reports a relationship with Bayer that includes: speaking and lecture fees. Yohei Sotomi reports a relationship with Boehringer Ingelheim that includes: speaking and lecture fees. Yohei Sotomi reports a relationship with Daiichi Sankyo Inc that includes: speaking and lecture fees. Yohei Sotomi reports a relationship with Pfizer Pharmaceuticals that includes: speaking and lecture fees. Yasushi Sakata reports a relationship with Bristol-Myers Squibb Company that includes: funding grants and speaking and lecture fees. Yasushi Sakata reports a relationship with Bayer AG that includes: funding grants and speaking and lecture fees. Yasushi Sakata reports a relationship with Boehringer Ingelheim that includes: funding grants and speaking and lecture fees. Yasushi Sakata reports a relationship with Pfizer that includes: speaking and lecture fees. Yasushi Sakata reports a relationship with Daiichi Sankyo that includes: speaking and lecture fees. If there are other authors, they declare that they have no known competing financial interests or personal relationships that could have appeared to influence the work reported in this paper.
