# Supplementary material for: Add-on antiplatelet therapy in anticoagulated patients with atrial fibrillation
Source: Int J Cardiol Heart Vasc. 2026 Mar 20;64:101907. doi: 10.1016/j.ijcha.2026.101907 (PMC13018862; doi:10.1016/j.ijcha.2026.101907)

**Supplemental Appendix**

**Table S1, S2, S3, S4**

**Figure S1, S2, S3, S4**

**Supplemental Table 1. Distribution of IPTW Weights and Effective Sample Size Under Alternative Weighting Specifications**

| spec | min | p1 | p50 | p99 | max | proportion truncated | ESS overall | ESS OAC+APT | ESS OAC alone |
| --- | --- | --- | --- | --- | --- | --- | --- | --- | --- |
| Unsterilized (primary) | 1.005001 | 1.021041 | 1.102521 | 16.08529 | 64.50299 |  | 2066.112 | 545.2013 | 5187.26 |
| Stabilized | 0.190575 | 0.221036 | 0.890329 | 3.691264 | 11.27296 |  | 5409.582 | 545.2013 | 5187.26 |
| Stabilized + truncation (1st/99th pct) | 0.221036 | 0.22106 | 0.890329 | 3.691175 | 3.691264 | 0.020035 | 5797.883 | 649.0084 | 5306.85 |

IPTW, inverse probability of treatment weighting; ESS, effective sample size. Values summarize the distribution of IPTW weights (min, 1st percentile [p1], median [p50], 99th percentile [p99], max). “Proportion truncated” indicates the fraction of observations whose weights were truncated at the 1st/99th percentiles. ESS was calculated for the overall sample and within each treatment group.

**Supplemental Table 2. Sensitivity analyses for the primary endpoints.**

Sensitivity analyses included (a) alternative IPTW specifications using stabilized weights with truncation at the 1st/99th percentiles, (b) an alternative ischemic composite excluding myocardial infarction, and (c) exclusion of the SAKURA-AF registry. Values are weighted hazard ratios (wHR) with 95% confidence intervals.

|  | Primary ischemic endpoint | | | Primary bleeding endpoint | | |
| --- | --- | --- | --- | --- | --- | --- |
|  | wHR | 95% CI | P value | wHR | 95% CI | P value |
| Primary analysis | 1.28 | 1.17 - 1.40 | <0.001 | 1.26 | 1.19 - 1.33 | <0.001 |
| 1. Stabilized + truncation (1st/99th percentile) | 1.34 | 1.14 - 1.57 | <0.001 | 1.28 | 1.15 - 1.42 | <0.001 |
| 1. Alternative ischemic composite excluding MI | 1.15 | 1.04 - 1.27 | 0.008 |  |  |  |
| 1. Excluding SAKURA-AF registry | 1.18 | 0.98 -1.28 | 0.107 | 1.27 | 1.19 - 1.35 | <0.001 |

**Supplemental Table 3. Baseline Characteristics of Study Population after Inverse Probability of Treatment Weighting, Stratified by vascular disease status**

|  | Vascular disease | |  | Nonvascular disease | |  |
| --- | --- | --- | --- | --- | --- | --- |
|  | OAC alone | OAC + APT | SMD | OAC alone | OAC + APT | SMD |
| Weighted N | 1263 | 1265 |  | 6123 | 6127 |  |
| Age | 75.0 [69.0, 82.0] | 76.0 [70.0, 81.0] | 0.014 | 72.0 [65.0, 78.0] | 72.0 [65.0, 78.0] | 0.092 |
| Female | 350 (27.7) | 349 (27.6) | 0.002 | 1951 (31.9) | 2033 (33.2) | 0.028 |
| Body mass index, kg/m2 | 23.6 [20.9, 26.2] | 23.7 [21.1, 26.6] | 0.002 | 23.5 [21.0, 26.2] | 23.5 [21.3, 26.0] | 0.040 |
| Creatinine clearance, mL/min | 56.5 [44.9, 74.4] | 58 [42.8, 74.3] | 0.001 | 66.3 [50.6, 85.0] | 65.9 [50.2, 86.4] | 0.037 |
| History of stroke or transient ischemic attack | 337 (26.7) | 338 (26.7) | 0.001 | 996 (16.3) | 991 (16.2) | 0.003 |
| Hypertension | 1050 (83.1) | 1053 (83.2) | 0.003 | 4031 (65.8) | 3867 (63.1) | 0.057 |
| Diabetes | 503 (39.8) | 497 (39.2) | 0.012 | 1282 (20.9) | 1328 (21.7) | 0.018 |
| Dyslipidemia | 863 (68.3) | 864 (68.3) | <0.001 | 2589 (42.3) | 2695 (44.0) | 0.034 |
| History of bleeding | 215 (17.0) | 215 (17.0) | <0.001 | 435 (7.1) | 583 (9.5) | 0.087 |
| Liver dysfunction | 259 (20.5) | 262 (20.7) | 0.004 | 737 (12.0) | 753 (12.3) | 0.008 |
| Type of OAC |  |  | 0.012 |  |  | 0.111 |
| Dabigatran | 194 (15.3) | 198 (15.7) |  | 1148 (18.7) | 1191 (19.4) |  |
| Rivaroxaban | 299 (23.7) | 301 (23.8) |  | 1570 (25.6) | 1318 (21.5) |  |
| Apixaban | 377 (29.8) | 372 (29.4) |  | 1312 (21.4) | 1351 (22.0) |  |
| Edoxaban | 200 (15.9) | 201 (15.9) |  | 726 (11.9) | 875 (14.3) |  |
| Warfarin | 193 (15.3) | 194 (15.4) |  | 1367 (22.3) | 1392 (22.7) |  |
| Hemoglobin, g/dL | 13.1 [11.8, 14.4] | 13.2 [11.8, 14.3] | 0.014 | 13.8 [12.5, 14.9] | 13.6 [12.2, 15.0] | 0.049 |
| Platelet, 10^3^/µL | 187.3 [159.0, 219.0] | 187.0 [155.0, 222.0] | 0.002 | 196.0 [163.0, 230.0] | 193.0 [161.4, 229.0] | 0.026 |
| Registry |  |  | 0.003 |  |  | 0.097 |
| SAKURA-AF | 374 (29.6) | 377 (29.8) |  | 2865 (46.8) | 2838 (46.3) |  |
| DIRECT registry | 601 (47.6) | 600 (47.5) |  | 1868 (30.5) | 1670 (27.3) |  |
| Osaka University Hospital | 288 (22.8) | 288 (22.8) |  | 1391 (22.7) | 1619 (26.4) |  |

Data are expressed as median [IQR] or number (percentage). OAC alone group: treated with oral anticoagulants alone; OAC+APT group: treated with both oral anticoagulants and antiplatelet therapy. Weighted N represents the sum of IPTW weights (pseudo-population), not the number of observed patients. Abbreviations; SMD, standardized mean difference; OAC, oral anticoagulants

**Supplemental Table 4. Clinical impact of add-on antiplatelet therapy stratified by vascular disease status**

|  | Vascular disease (+) | | | | | Vascular disease (-) | | | | |  |
| --- | --- | --- | --- | --- | --- | --- | --- | --- | --- | --- | --- |
|  | Event number/ Event rate (/100 person-yeasr) | |  |  |  | Event number/ Event rate (/100 person-years) | |  |  |  |  |
|  | OAC alone  N = 532 | OAC+APT  N = 732 | wHR | 95% CI | P value | OAC alone  N=5,564 | OAC+APT  N=559 | wHR | 95% CI | P value | P value for interaction |
| Primary ischemic endpoint (a composite of all-cause death, ischemic stroke, systemic embolism, and myocardial infarction) | 92/6.44 | 142/6.99 | 0.96 | 0.80 - 1.15 | 0.664 | 543/3.61 | 89/5.82 | 1.38 | 1.24 - 1.53 | <0.001 | <0.001 |
| All-cause death | 57/3.87 | 78/3.68 | 0.84 | 0.67 - 1.06 | 0.148 | 287/1.85 | 45/2.80 | 1.24 | 1.07 - 1.43 | 0.005 | <0.001 |
| Ischemic stroke | 23/1.57 | 23/1.10 | 0.63 | 0.42 - 0.96 | 0.031 | 146/0.96 | 23/1.46 | 1.27 | 1.04 - 1.55 | 0.022 | <0.001 |
| Systemic embolism | 8/0.54 | 20/0.95 | 1.97 | 1.08 - 3.60 | 0.028 | 54/0.35 | 8/0.50 | 1.29 | 0.93 - 1.79 | 0.131 | 0.360 |
| Myocardial infarction | 24/1.65 | 47/2.28 | 1.17 | 0.84 - 1.64 | 0.347 | 183/1.20 | 34/2.17 | 1.77 | 1.49 - 2.10 | <0.001 | 0.012 |
| Primary bleeding endpoint (Any bleeding) | 233/24.12 | 317/23.07 | 0.99 | 0.88 - 1.11 | 0.866 | 1561/12.94 | 207/18.22 | 1.33 | 1.25 - 1.41 | <0.001 | <0.001 |
| Major bleeding | 56/4.05 | 80/3.98 | 1.03 | 0.81 - 1.31 | 0.807 | 350/2.33 | 53/3.47 | 1.10 | 0.96 - 1.26 | 0.172 | 0.246 |
| Clinically relevant non-major bleeding | 218/21.91 | 294/20.90 | 0.99 | 0.87 - 1.12 | 0.840 | 1395/11.35 | 190/16.35 | 1.37 | 1.29 - 1.47 | <0.001 | <0.001 |

Event rates are crude incidence rates per 100 person-years (unweighted). wHRs were estimated using IPTW-weighted Cox models. P for interaction was derived from a treatment-by-vascular disease interaction term. Abbreviations: wHR, weighted hazard ratio; CI, confidence interval.

**Supplemental Figure 1. Study flowchart**

Out of 7,512 patients, after excluding 125 patients because they received PCI or CABG within 1 year, 7,387 patients were finally eligible. These patients were divided into two groups by concomitant use of antiplatelets; OAC alone group (treated with OAC monotherapy; N = 6,096) and OAC+APT group (treated with both OAC and antiplatelet therapy; N = 1,291). Abbreviations: AF, atrial fibrillation; APT, antiplatelets; PCI, percutaneous coronary intervention; CABG, coronary artery bypass grafting; OAC, oral anticoagulant.


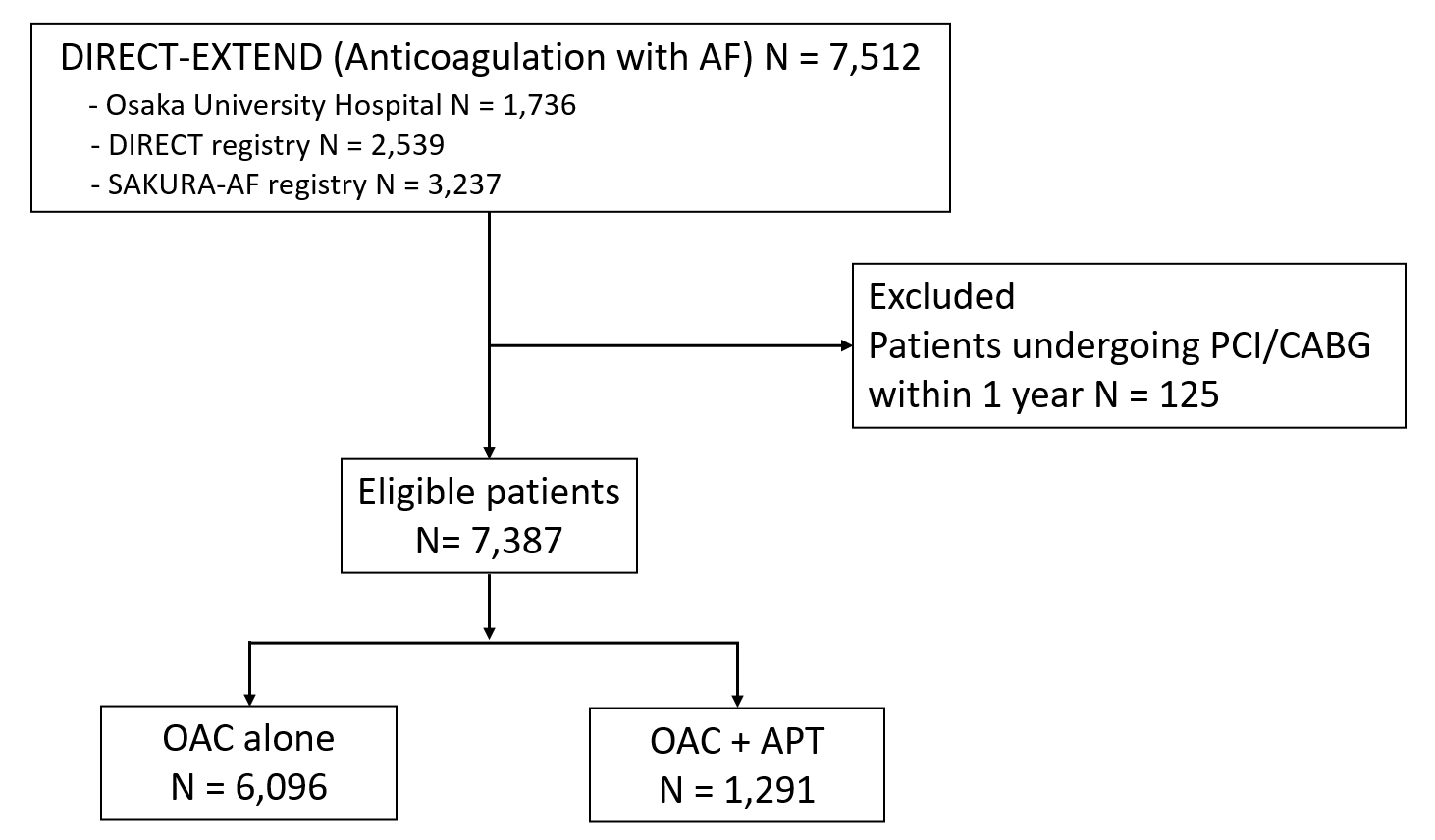


**Supplemental Figure 2. Distributions of propensity scores and inverse probability of treatment weights.**

Panel A shows the distribution of propensity scores by treatment group. Propensity scores were estimated using a multivariable logistic regression model including age, female sex, body mass index, creatinine clearance, history of stroke or transient ischemic attack, vascular disease, diabetes mellitus, hypertension, dyslipidemia, history of bleeding, liver dysfunction, type of OAC, hemoglobin, platelet count, and the registry the patient was included in. Panel B shows the distribution of unstabilized inverse probability of treatment weights used in the primary IPTW analysis. For visualization, Panel B is limited to weights up to the 99th percentile.

**
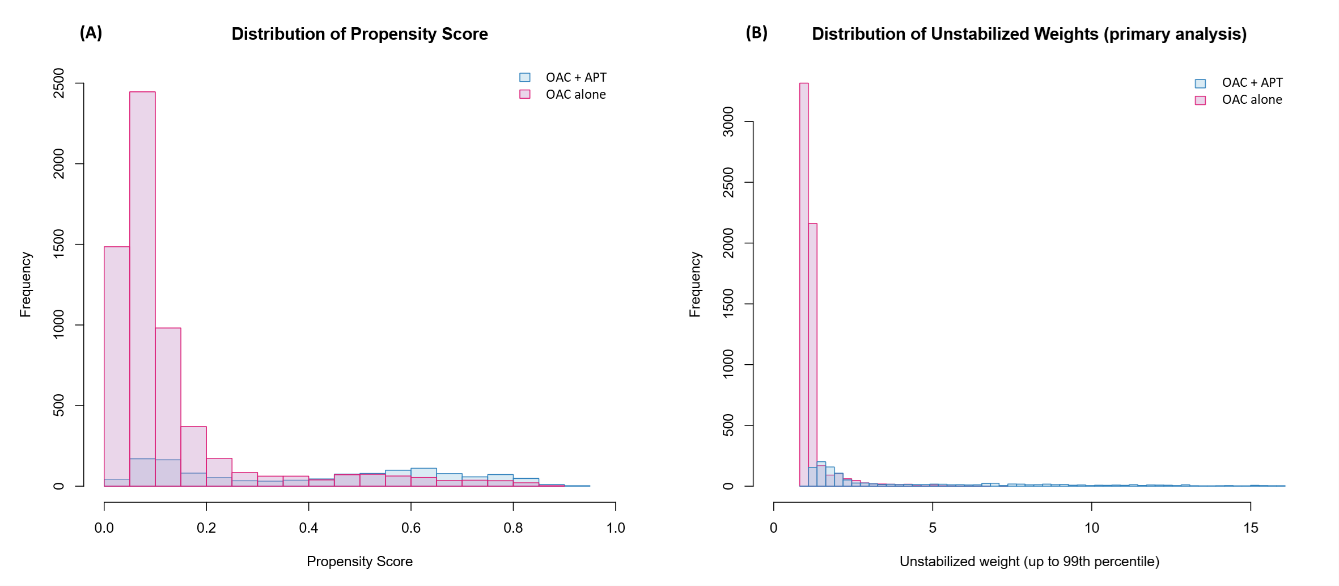
**

**Supplemental Figure 3. Days from bleeding event to ischemic event**

Patients who experienced both bleeding and ischemic events were extracted from the entire cohort. The time interval between the bleeding event and the subsequent ischemic event was calculated and displayed as a histogram. Each bar on the horizontal axis represents a 30-day interval.


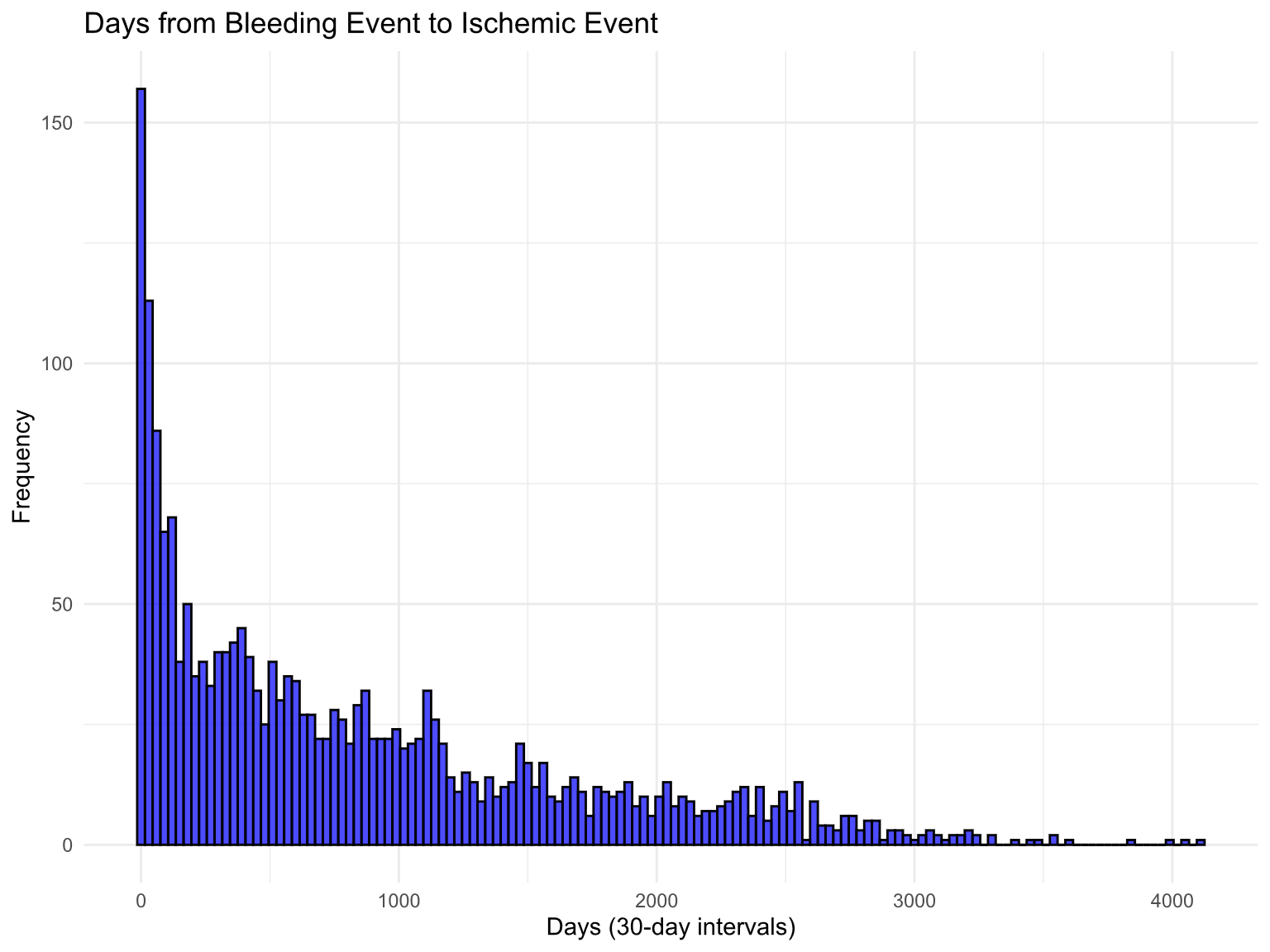


**Supplemental Figure 4. Kaplan-Meier curves stratified by vascular disease**

Survival analysis using the Kaplan-Meier method for patients with (A) vascular disease and (B) non-vascular disease. Among patients with vascular disease, the risk of both ischemic and bleeding endpoints was similar between the OAC-alone group and the OAC+APT group (both log-rank p = 0.60). In contrast, among patients without vascular disease, those in the OAC+APT group demonstrated a significantly higher risk for both the primary ischemic and bleeding endpoints compared to the OAC-alone group (p < 0.001 for both endpoints). OAC alone group: treated with oral anticoagulants alone; OAC+APT group: treated with both oral anticoagulants and antiplatelet therapy.


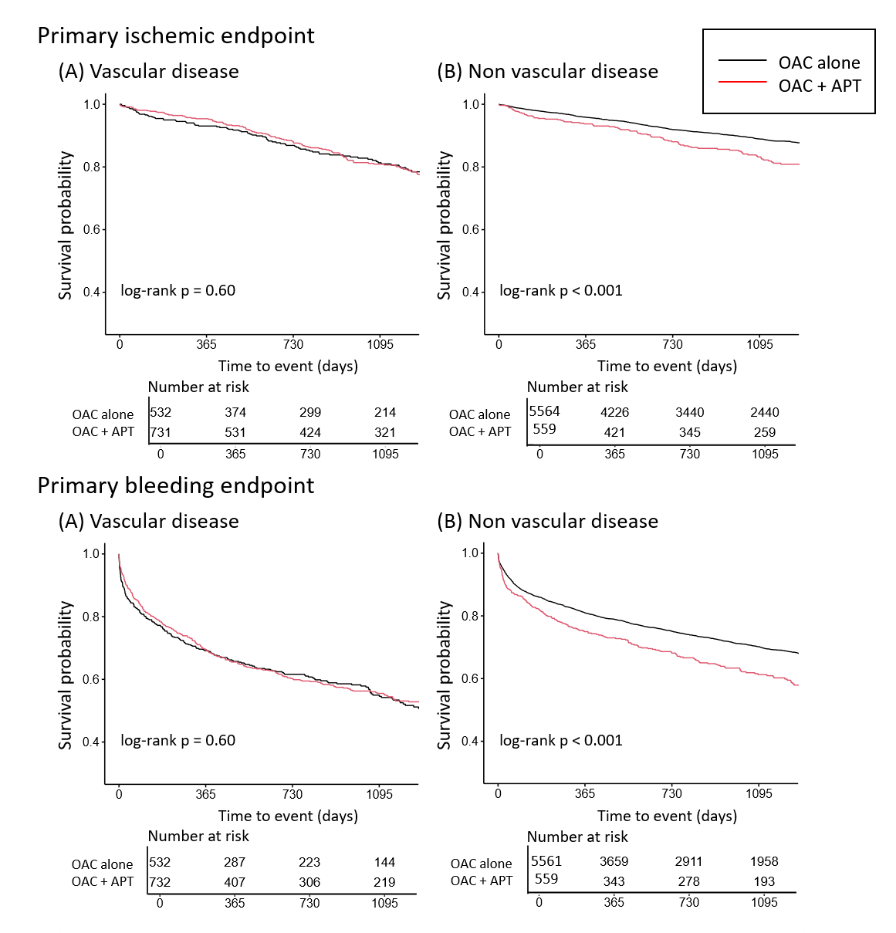

Supplement: Supplementary Data 1 [file mmc1.docx]
